# Synthesis, Characterization of Low Molecular Weight Chitosan Selenium Nanoparticles and Its Effect on DSS-Induced Ulcerative Colitis in Mice

**DOI:** 10.3390/ijms232415527

**Published:** 2022-12-08

**Authors:** Shu-Jiang Peng, Da-Tian Ye, Jie Zheng, Ya-Ru Xue, Lin Lin, Ya-Dong Zhao, Wen-Hua Miao, Yan Song, Zheng-Shun Wen, Bin Zheng

**Affiliations:** Zhejiang Provincial Engineering Technology Research Center of Marine Biomedical Products, School of Food Science and Pharmaceutics, Zhejiang Ocean University, Zhoushan 316022, China

**Keywords:** selenium nanoparticles, LCS-SeNPs, chitosan, characterization, colitis

## Abstract

Selenium nanoparticles have attracted extensive attention due to their good bioavailability and activity. In the present study, a new form of selenium nanoparticle (Low molecular weight chitosan selenium nanoparticles (LCS-SeNPs)) were synthesized in a system of sodium selenite and acetic acid. The size, element state, morphology and elementary composition of LCS-SeNPs were characterized by using various spectroscopic and microscopic measurements. The protection of LCS-SeNPs against dextran sulfate sodium (DSS)-induced intestinal barrier dysfunction and the inherent mechanisms of this process were investigated. The results showed that LCS-SeNPs, with an average diameter of 198 nm, zero-valent and orange-red relatively uniform spherical particles were prepared. LCS-SeNPs were mainly composed of C, N, O and Se elements, of which Se accounted for 39.03% of the four elements C, N, O and Se. LCS-SeNPs reduced colon injury and inflammation symptoms and improved intestinal barrier dysfunction. LCS-SeNPs significantly reduced serum and colonic inflammatory cytokines TNF-α and IL-6 levels. Moreover, LCS-SeNPs remarkably increased antioxidant enzyme GSH-Px levels in serum and colonic tissue. Further studies on inflammatory pathways showed that LCS-SeNPs alleviated DSS-induced colitis through the NF-κB signaling pathway, and relieved inflammatory associated oxidative stress through the Nrf2 signaling pathway. Our findings suggested that LCS-SeNPs are a promising selenium species with potential applications in the treatment of oxidative stress related inflammatory intestinal diseases.

## 1. Introduction

Inflammatory bowel disease (IBD) is a chronic inflammatory problem of the gastrointestinal tract, including Crohn’s disease (CD), ulcerative colitis (UC) and other diseases [1]. Its prevalence is increasing year by year, especially in Europe and North America. It is reported that the prevalence of Crohn’s disease is 1522–21,312 cases/100,000 people, and the prevalence of UC is 2422–2946 cases/100,000 people [2]. Recent studies have shown that the pathogenesis of IBD may be related to genetics, intestinal microbes, environmental factors and immune abnormalities [3].

Chitosan is the only natural polysaccharide with a positive charge, that is obtained by deacetylation of naturally occurring chitin from seafood processing wastes. It is broadly found in insects, fungi and the exoskeletons of crustaceans [4]. It is broadly utilized as a carrier for an assortment of drugs due to its low toxicity and capacity to upgrade medicated bioadhesion and bioavailability [5,6]. Low molecular weight chitosan are the products of chitosan degradation and they have antibacterial, anti-inflammatory, antioxidant, anticancer and immunomodulatory activities [7,8,9].

Selenium is an indispensable trace element for human beings, showing a variety of biological functions [10]. The intake dosage of selenium in adult males and females is 55 μg and 70 μg per day, respectively [11]. Selenium is important for human health as it is an important component of several major metabolic pathways, including thyroid hormone metabolism [12,13], antioxidant defense systems and immune function [14]. Previous studies have shown that selenium plays a positive role in inducing apoptosis of cancer cells [15,16], for example, diminishing the frequency of lung cancer, prostate cancer and other cancers [17,18]. In addition, selenium has strong antioxidant, immune and anti-inflammatory properties. The main source of selenium intake is through food, but the organic selenium content in food is very low and is insufficient for most people [19,20]. Selenium deficiency can lead to chronic inflammatory diseases, such as cardiovascular disease and inflammatory bowel disease (IBD) [21]. IBD is characterized by the hyperstimulation of the colon and the little digestive tract, counting Crohn’s malady (CD) and ulcerative colitis (UC). Low selenium levels were observed in both UC and CD patients [22]. In addition, low selenium status was found to be associated with increased CD severity and colon cancer risk and was associated with epithelial injury [23,24]. It has been found that Mannose-rich Oligosaccharides-functionalized selenium nanoparticles revere inflammation in UC by accelerating the reprogramming of macrophages [25].

Nanotechnology is a comprehensive technology with strong cross-cutting, and its research covers a wide range of fields, including physical and chemical materials and medicine [26,27,28]. General nutritional supplements are insufficient in solubility, bioavailability, permeability and stability, which limits the role of supplements [29]. The combination of nanotechnology and nutritional supplements can solve the shortcomings of traditional supplements and ensure their biological activity [30,31]. Selenium is an essential element for human life, and it is widely used as a dietary supplement [32]. Elemental selenium powder in the redox state of 0 is unstable, not soluble and is generally considered to be biologically inert. However, nano-sized elemental selenium particles were found to possess higher activity, bioavailability and lower toxicity [33]. Studies have found that selenium nanoparticles play an important role in the immune system, nervous system diseases, tumors, diabetes, oxidative stress and inflammation [34,35,36,37,38], which are attracting increasing attention. The previous studies found that zero-valence selenium was capped with suitable carriers to improve its stability [39,40]. Particle size affects and influences the cellular admissions of nanoparticles (NPs), for example, in vitro absorption of 0.1 μm particles was found to be 2.5 and 6 times higher when compared to 1 and 10 μm particles, respectively [41]. It has also been found that nanoparticles with smaller particle size have a better free radical scavenging effect [42]. Therefore, the biological characteristics of selenium nanoparticles are related to their particle size. The SeNPs, prepared with polysaccharides as a stabilizer and a dispersant, have a higher stability, bioavailability and bioactivity than single polysaccharides or sodium selenite [43,44]. Tang et al. found that the nano-selenium (Nano-Se) particles, modified by Gracilaria lemaneiformis polysaccharides, have a higher antioxidant activity than the single Gracilaria lemaneiformis polysaccharidese and sodium selenite [45]. However, to our knowledge, there are few studies on nano selenium particles modified by low molecular weight chitosan, as most of them use chitosan to stabilize selenium nanoparticles, studying their activities in antitumor, diabetes, antioxidant, antibacterial and brain damage [46,47,48,49]; however, its mechanism of action in alleviating colitis in vivo is still unclear.

In the present study, LCS-SeNPs were developed using low molecular weight chitosan as a dispersant and a stabilizer and were characterized by X-ray photoelectron spectroscopy (XPS), energy-dispersive X-ray spectroscopy (EDX) and transmission electron microscopy (TEM). Moreover, the effect of LCS-SeNPs on intestinal oxidative stress and permeability were evaluated by measuring the levels of TNF-α, IL-6, GSH-Px, LPS and DAO in the serum of mice with DSS-induced colitis. The effect of LCS-SeNPs on the DSS-induced inflammatory response and intestinal barrier injury were investigated via detecting levels of TNF-α, IL-6 and tight junction proteins, including occludin and ZO-1 in the colon using RT-PCR. The underlying mechanism of LCS-SeNPs in preventing DSS-induced ulcerative colitis was also studied by Western blot analysis in this study.

## 2. Results

### 2.1. Characterization of LCS-SeNPs

#### 2.1.1. Valence State of Selenium

**Figure 1:**
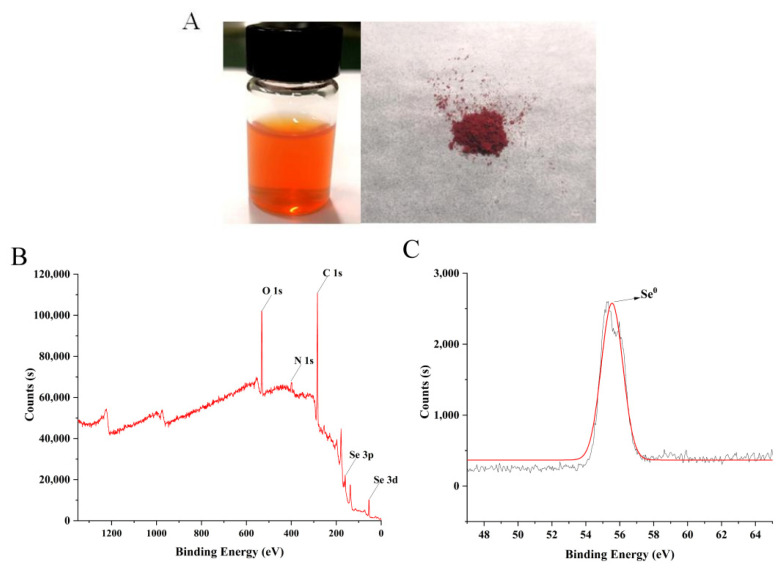
The solution and lyophilized powder of LCS-SeNPs (**A**); XPS spectrum of LCS-SeNPs (**B**,**C**).

As shown in Figure 1A, the solution of LCS-SeNPs appears orange-red and after freeze-drying it becomes a red powder, indicating that red elemental nano-selenium is generated. The valence state of selenium in LCS-SeNPs was detected by XPS (Figure 1B,C). The orbital signals of O1s, N1s, C1s, Se3p and Se3d were detected in the XPS energy spectrum, the 3D energy spectrum peak of Se0 is shown in Figure 1C, and the binding energy of the Se 3D spectra of LCS-SeNPs is 55.39 eV, which is consistent with the previous studies [50,51]. This indicated that Se within the LCS-SeNPs was in an elementary state. In addition, there is no typical Se 3D signal with 59.5 eV belonging to Se (IV) in the spectrum, confirming that Se (IV) was completely reduced to red elemental selenium (Se0) in the preparation process [27].

#### 2.1.2. Particle Size and Morphology of LCS-SeNPs

**Figure 2:**
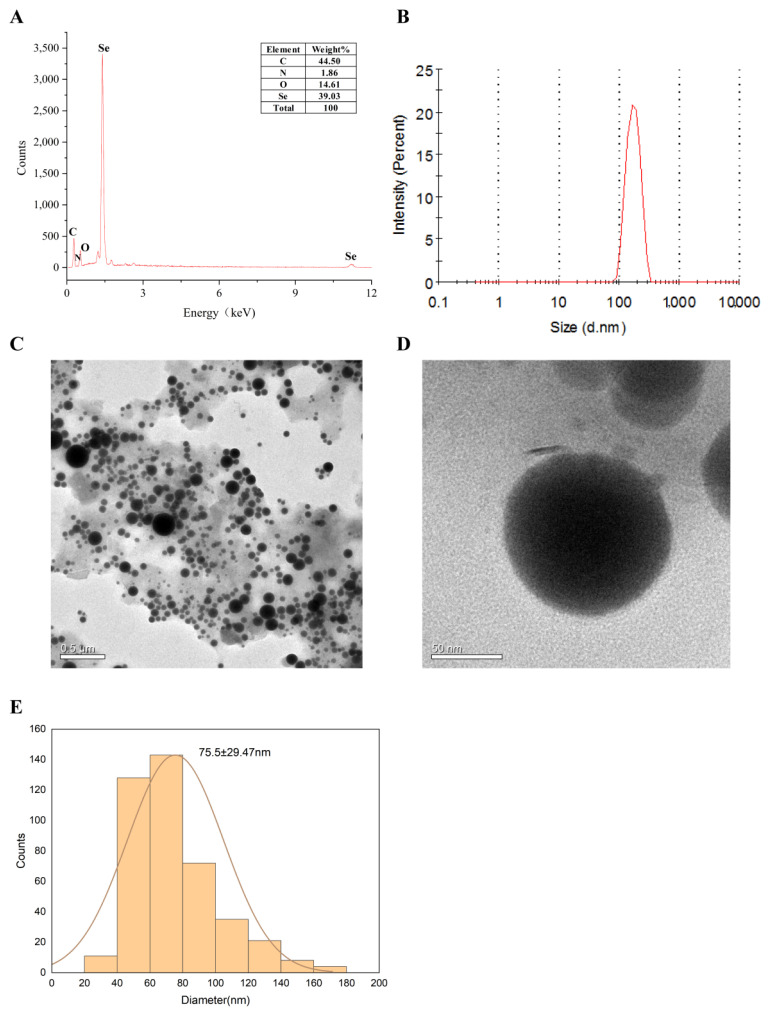
EDX spectrum (**A**); Particle size distribution diagram (**B**); TEM diagram of LCS-SeNPs (**C**,**D**); Size distribution of LCS-SeNPs (**E**).

The morphology of LCS-SeNPs was observed using the transmission electron microscope (TEM), as shown in Figure 2C,D, In the case of low molecular weight chitosan as a dispersant and stabilizer, the nano-selenium particles did not form into a cluster polymerization but formed relatively dispersed nano-selenium particles with a small particle size.

The particle size of LCS-SeNPs was investigated by using a Zetasizer_Nano_ZS90 (Malvern Instruments Limited, Malvern, UK) particle analyzer, as shown in Figure 2B. The average diameter of LCS-SeNPs were 198 nm; moreover, LCS-SeNPs have a narrow particle size distribution, which proved that the prepared LCS-SeNPs were relatively uniform. The element composition of LCS-SeNPs was analyzed by EDX, as shown in Figure 2A. The Se element accounted for 39.03% of the four elements C, N, O and Se.

### 2.2. LCS-SeNPs Altered the Physical Signs and Colon Histopathological Scores in DSS-Induced Colitis Mice

**Figure 3:**
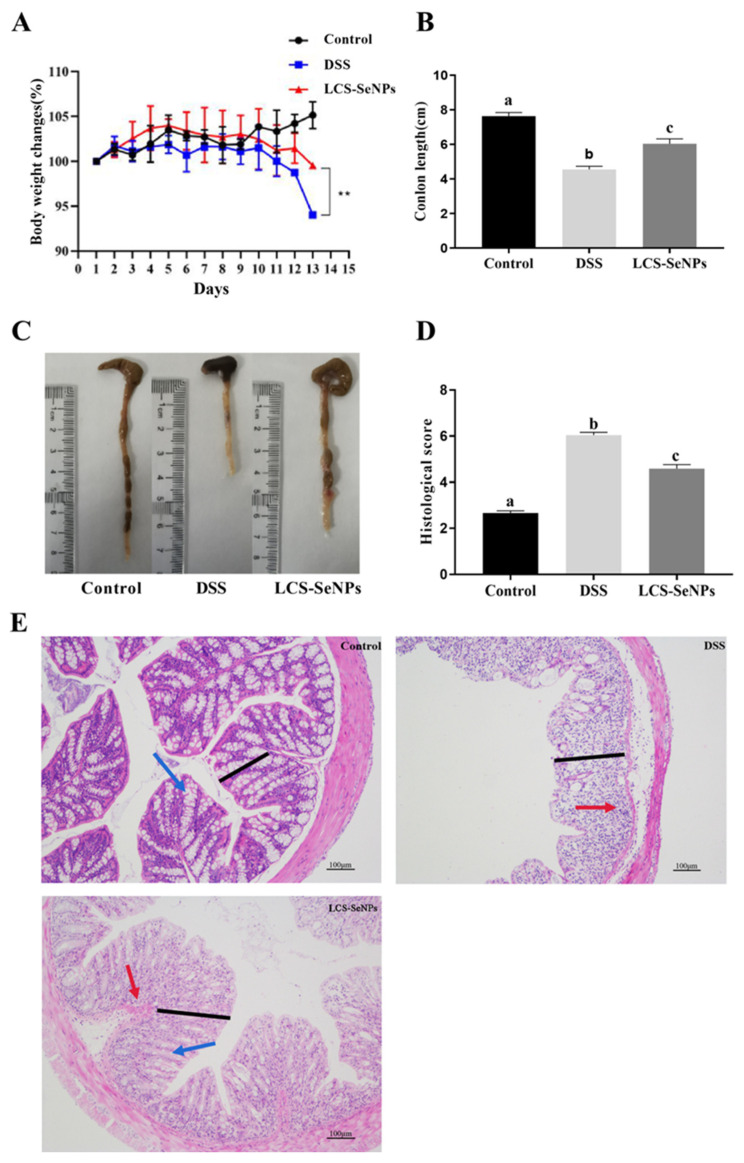
LCS-SeNPs alleviates DSS-induced colitis. Body weight change in mice (**A**); colon length in different groups (**B**); images of colon length from each group of mice (**C**); histological score (**D**); H&E staining images of each group, black line segment indicates mucosal layer, blue arrow indicates goblet cells and red arrow indicates inflammatory cells (**E**). Data are presented as mean ± SEM. In the same column values with different letters superscripts differ significantly (** *p* < 0.01).

The alleviating effect of LCS-SeNPs on DSS-induced colitis in mice was investigated. As shown in Figure 3A, the weight of mice in the DSS treatment increased significantly (*p* < 0.05) when compared with the control group. The length of the colon became shorter (*p* < 0.05) after DSS treatment when compared with the control group (Figure 3B,C). However, the LCS-SeNPs significantly improved the shortening of the length of the mouse colon induced by DSS, which proved that LCS-SeNPs had a certain alleviating effect on mouse colitis.

As indicated by the H&E staining results, the colon tissue of the DSS group of mice showed damage to the colonic histopathology, including damage to the mucosal layer, with a large number of goblet cells disappearing, and inflammatory cells seriously infiltrating. While the pathology phenomenon was significantly alleviated, the mucosal layer was intact; however, a small part of the goblet cells disappeared, and the inflammatory cell infiltration was alleviated after LCS-SeNPs treatment. Quantitative results of the colon histopathological score showed that LCS-SeNPs can significantly (*p* < 0.05) reduce the histological score of colitis, as shown in Figure 3D,E.

### 2.3. Effects of LCS-SeNPs on Serum Inflammatory Cytokines Levels and Antioxidant Capacity of DSS-Induced Colitis Mice

**Figure 4:**
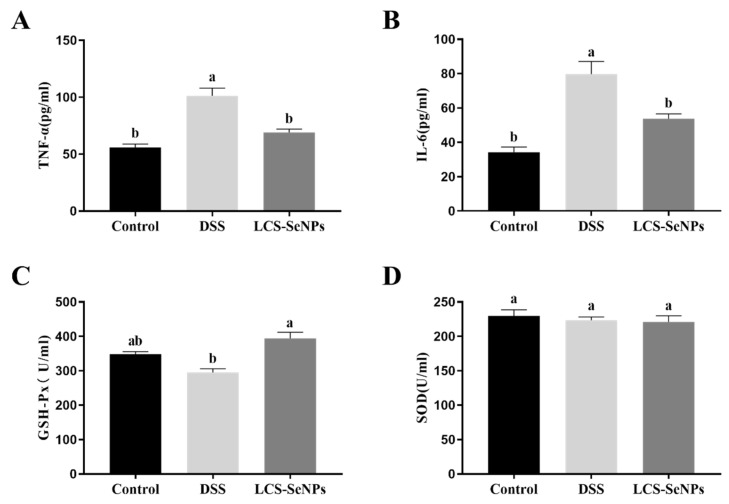
Effects of LCS-SeNPs supplementation on serum inflammation cytokine and antioxidant parameters in DSS-induced colitis mice. The serum inflammation cytokine levels of TNF-α (**A**) and IL-6 (**B**). The levels of GSH-Px (**C**) and SOD (**D**). Data are presented as mean ± SEM. In the same column values with different letters superscripts differ significantly (*p* < 0.05).

Serum IL-6 and TNF-α were detected as pro-inflammatory cytokines. DSS treatment significantly (*p* < 0.05) increased the levels of pro-inflammatory cytokine TNF-α and IL-6 when compared with the control group, while LCS-SeNPs significantly decreased (*p* < 0.05) TNF-α and IL-6 levels (Figure 4A,D). When compared to the control group, the DSS group had a lower serum level of GSH-Px (*p* < 0.05), while the serum level of GSH-Px in LCS-SeNPs group increased (*p* < 0.05), but there was no significant difference between the DSS group and the LCS-SeNPs group. There was no significant difference in the SOD levels in the serum of the three groups of mice, as shown in Figure 4D.

### 2.4. LCS-SeNPs Altered Intestinal Permeability in DSS-Induced Colitis Mice

**Figure 5:**
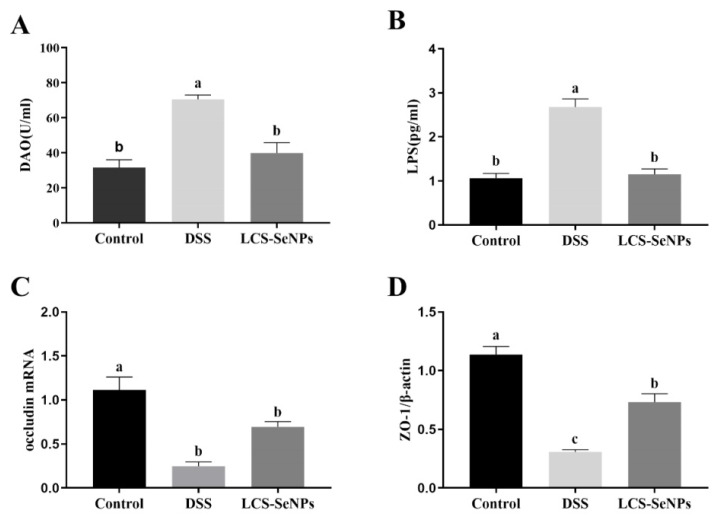
LCS-SeNPs altered intestinal permeability in DSS-induced colitis mice. The levels of DAO (**A**) and LPS (**B**) in serum; the mRNA expression levels of occludin (**C**) and ZO-1 (**D**) in colon tissue. Data are presented as mean ± SEM. In the same column values with different letters superscripts differ significantly (*p* < 0.05).

DSS treatment significantly increased DAO and LPS levels when compared with the control group, while LCS-SeNPs treatment reversed this effect, leaving no significant difference in DAO and LPS levels between the control group and mice, as shown in Figure 5A,B.

Furthermore, the mRNA expression levels of occludin and ZO-1, related to intestinal permeability, were detected in the colon. The DSS group showed a remarkable decrease in colonic occludin and ZO-1 mRNA levels when compared with the control group, while the mRNA expression levels of occludin and ZO-1 were significantly increased after LCS-SeNPs treatment (Figure 5C,D).

### 2.5. Effects of LCS-SeNPs on Inflammatory Factors and Antioxidant Capacity of Colon Tissue in DSS-Induced Colitis Mice

**Figure 6:**
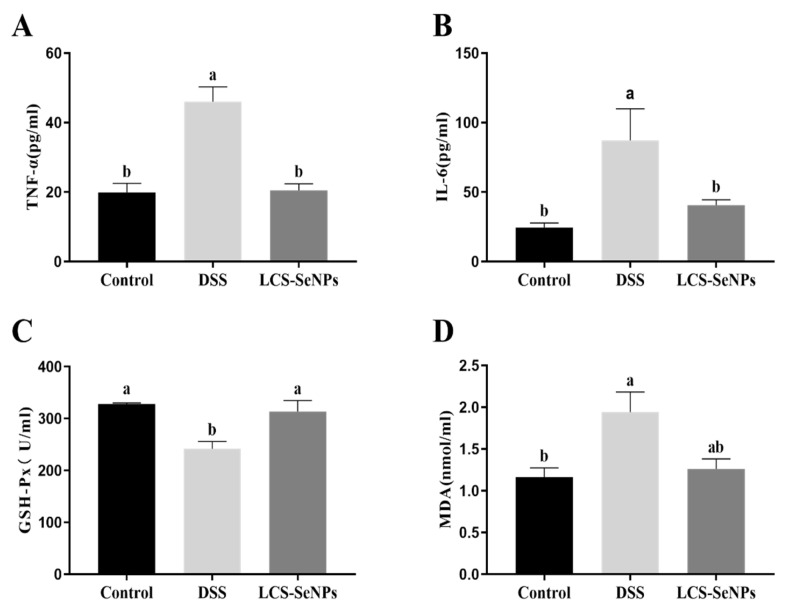
Effects of LCS-SeNPs supplementation on colon tissue inflammation cytokine and antioxidant parameters in DSS-induced colitis mice. The inflammation cytokine levels of TNF-α (**A**) and IL-6 (**B**) in colon tissue; the levels of GSH-Px (**C**) and MAD (**D**) in colon tissue. Data are presented as mean ± SEM. In the same column values with different letters superscripts differ significantly (*p* < 0.05).

Inflammatory cytokines (IL-6 and TNF-α) levels were significantly (*p* < 0.05) increased in the colon tissue of DSS-induced colitis mice when compared with the control group, while LCS-SeNPs significantly reduced (*p* < 0.05) the levels of pro-inflammatory cytokines IL-6 and TNF (Figure 6A,B). The enzyme activity of GSH-Px in the DSS group was significantly decreased when compared with the control group and the LCS-SeNPs group, as shown in Figure 6C,D. The MDA levels in the DSS group were significantly (*p* < 0.05) higher than those in the control group.

### 2.6. Effects of LCS-SeNPs on the Nrf-2 Signaling Pathways in DSS-Induced Colitis Mice

**Figure 7:**
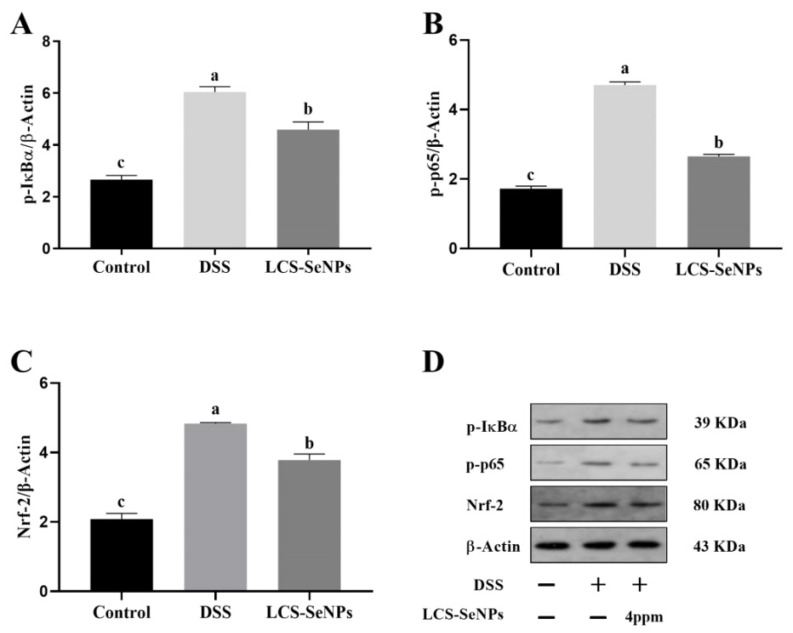
LCS-SeNPs down-regulated p-IκBα, p-p65 and Nrf-2 expression in DSS-induced Colitis. The relative protein expression of p-IκBα (**A**); p-p65 (**B**) and Nrf-2 (**C**) were normalized to β-actin. Representative immunoblot results of p-IκBα, p-p65 and Nrf-2 in the colon (**D**). Data are presented as mean ± SEM. In the same column values with different letters superscripts differ significantly (*p* < 0.05).

A significant increase (*p* < 0.05) in the expression levels of p-IκBα and p-p65 was observed in the DSS group, while the expression levels of p-IκBα and p-p65 were significantly decreased (*p* < 0.05) in the LCS-SeNPs group (Figure 7A,B). In addition, as shown in Figure 7C, LCS-SeNPs can reduce the increased expression of Nrf-2 caused by DSS, thus reducing the intestinal damage caused by oxidative stress.

## 3. Discussion

Selenium is an indispensable trace element in organisms, showing a variety of biological activities, but it has cytotoxicity [52]. Therefore, it is important to pay attention to not only retain the activity of selenium but also improve its safety. Previous studies have demonstrated that natural polysaccharides and their degraded oligosaccharides have a variety of biological activities, including anti-inflammatory, anticancer and immune activities [53,54,55]. Nano selenium is a form of selenium, which has a lower toxicity and a higher utilization rate in organisms [56,57]. Previous studies have shown that there is a synergistic effect between natural polysaccharides and nano-selenium [28]. Many studies have proved that nano-selenium, prepared with polysaccharides as a dispersant and a stabilizer, exhibits a strong activity, which is stronger than that of polysaccharides [27,57,58], and its toxicity is lower than that of sodium selenite.

Changes in the intestinal permeability are important influencing factors in the occurrence and development of colitis. DAO and LPS are closely related to the intestinal barrier, and an impaired intestinal barrier function will lead to increased levels of DAO and LPS in serum [53,59]. Present findings showed that LCS-SeNPs significantly reduced the levels of DAO and LPS in serum, and significantly increased the expression levels of occludin and ZO-1 genes, suggesting that LCS-SeNPs have a protective effect on the functional impairment of the intestinal barrier caused by DSS. In inflammatory bowel disease (IBD), such as ulcerative colitis or Crohn’s disease, the intestinal epithelium is easily destroyed during intestinal inflammation. IBD can cause disruption of the integrity of the epithelial barrier through dysfunction of the molecular circuits within the intestinal epithelial cells that control homeostasis, renewal and repair [45]. The barrier function is mainly reflected in three aspects: Mechanical barrier, which is composed of the close connection between intestinal cell membrane and intestinal cells. Symbiotic bacteria interact with host to form a microbial ecosystem as an ecological barrier. Intestinal associated lymphoid tissue and immune molecules establish an immune barrier [60]. In addition, ZO-1 and occludin are an important part of tight junctions and have a significant influence on intestinal permeability [61]. The present study suggested that DSS can promote the increase in intestinal permeability, while LCS-SENPs can significantly reduce the increase in intestinal permeability caused by DSS.

The antioxidant enzymes in gastrointestinal organs mainly play the role of antioxidant via eliminating free radicals and protecting the intestine from oxidative injury [62]. Intestines act as the largest immune system for the body and is easily attacked by excessive free radicals, inducing oxidative stress [63]. Nuclear transcription factor Nrf2, when activated, binds to antioxidant response elements (ARE) to induce the expression of its downstream phase II detoxification enzyme HO-1 [64] and antioxidant enzyme genes [65]. In the present study, we measured the levels of GSH-Px and MDA in the colon tissue of mice treated with DSS to investigate whether LCS-SeNPs affect oxidative stress. Our findings showed that the administration of LCS-SENPs increased the enzyme activity of serum and colonic GSH-Px and reduced colonic MDA production. Previous studies suggested that GSH-Px plays an important role in protecting the colon against oxidative damage [66,67]. Oxidative stress or cell damage is caused by free radicals and usually involves lipid peroxidation [68]. There is increasing evidence that many selenium nanoparticles, conjugated with polysaccharides, exhibit antioxidant properties and anti-inflammatory effects that regulate diseases, such as IBD, atherosclerosis and cancer [28,69]. The present study suggested that LCS-SeNPs treatment significantly improved the enzyme activity reduction in GSH-Px caused by DSS. Western blot analysis indicated that the DSS obviously increased the expression of Nrf2. These results indicated that DSS could improve the Nrf2 signaling pathway when compared with the control group, while LCS-SeNPs treatment significantly inhibited the expression of Nrf2 in the intestinal mucosa of DSS-induced colitis in mice. Taken together, our results suggested that DSS probably disturbed the balance of the oxidative and the anti-oxidative systems in intestinal mucosa, and induced oxidative injury involved in the Nrf2-dependent signaling pathway.

Cytokines include anti-inflammatory cytokines (IL-4 and IL-10) and pro-inflammatory cytokines (IL-1, IL-6 and TNF-α), which are mainly involved in regulating the proliferation and differentiation of immune cells, immune responses and inflammatory responses. Our results showed that LCS-SeNPs reduced the pro-inflammatory cytokines TNF-α and IL-6 levels in serum and the colon of DSS-induced colitis mice, suggesting that LCS-SeNPs can achieve anti-inflammatory effects by reducing the serum levels of pro-inflammatory factors TNF-α and IL-6. In addition, in the colitis model, elevated levels of pro-inflammatory cytokines (TNF-α, IL-1β and IL-6) were found to enhance the inflammatory cascade and cause intestinal tissue damage in UC patients [6]. Down-regulation or blocking of pro-inflammatory cytokine activity is effective in the treatment of IBD [70]. The NF-κB signaling pathway is associated with inflammation and immunity, and its activation is associated with IBD.

In the present study, we investigated the expression levels of p-IκBα,p-p65 and Nrf-2 proteins by Western blotting. Our findings indicated that LCS-SeNPs inhibited the activation of NF-κB in colonic tissue and reduced the phosphorylation of P65 by inhibiting the phosphorylation of IκBα. Therefore, LCS-SeNPs may reduce inflammation caused by DSS by inhibiting the NF-κB signaling. NF-κB plays a central role in regulating immune and inflammatory processes, making it a key target for new therapies for inflammatory diseases [71]. Prior to activation, NF-κB is combined with IκBα, an inhibitory protein that inactivates NF-κB in the cytoplasm. NF-κB is released from the cytoplasm and transferred to the nucleus by phosphorylation, ubiquitination and degradation of IκBα in response to various stimuli, including LPS and pro-inflammatory factors.

## 4. Materials and Methods

### 4.1. Materials and Reagents

Low molecular weight chitosan was bought from Golden-Shell Pharmaceutical Co., Ltd. (Zhejiang, China). Sodium selenite was purchased from Changsha Harlem Yu Chemical Technology Co., Ltd. (Hunan, China). Dextran sulphate sodium was bought from MP Biomedicals Co., Ltd. (Santa Ana, CA, USA). Phosphate buffered saline (PBS, PH 7.2–7.4) was bought from Phygene biotechnology Co., Ltd. (Fujian, China). Acetic acid purchased from Sinopharm. The kits for SOD, GSH-Px and MDA were obtained from Nanjing Jiancheng Bioengineering Institute (Nanjing, China). Enzyme-linked immunosorbent assay (ELISA) kits for TNF-α and IL-6 were purchased from Wuhan Boster Biological Technology Co., Ltd. (Hubei, China). The Diamine oxidase (DAO) ELISA Kit was purchased from SenBeiJia Biological Technology Co., Ltd. (Nanjing, China). The Endotoxin (LPS) ELISA Kit was purchased from Wuhan Cusabio Biotech Co., Ltd. (Hubei, China). The Agarose (Agarose D-5), Reverse transcription Kit (PrimeScriptTM II 1st Strand cDNA Synthesis Kit) and the Fluorescence quantitative kit SYBR^®^ Premix Ex TaqTM II (Tli RNaseH Plus) was purchased from Takara. The 2000 DNA Marker, RNA loading buffer, DNA 6×loading buffer, defat dried milk, TEMED and BCA Protein Assay kit were purchased from Shanghai Beyotime Biotechnology Co., Ltd. (Shanghai, China). Phospho-NF-κB p65 (Ser536) (93H1) Rabbit mAb, NF-κB p65 (D14E12) XP^®^ Rabbit mAb, Phospho-IκBα (Ser32/36) (5A5) Mouse mAb and IκBα (L35A5) Mouse mAb were obtained from Cell Signaling Technology (Boston, MA, USA). ColorMixed Protein Marker (11–180 KD) was purchased from Beijing Solarbio Science &Technology Co., Ltd. (Beijing, China). Goat anti-Rabbit IgG (H + L) was purchased from Thermo Scientific Pierce (Rockford, IL, USA). Rabbit Anti-Nrf2 Polyclonal Antibody was purchased from Abcam (Cambridgeshire, UK). The Mouse β-actin ELISA Kit was purchased from Santa Cruz Biotechnology company (Dallas, TX, USA). Other Chemical reagents were of analytical grade.

### 4.2. Preparation of LCS-SeNPs

Low molecular weight chitosan (1 g) was added to a conical bottle filled with 30 mL of 3% acetic acid solution and stirred for 1 h at room temperature to fully dissolve the low molecular weight chitosan. Subsequently, 10 mL sodium selenite solution (10 mg/mL) was added to the conical bottle, and then the mixture react in dark conditions under magnetic stirring for 48 h at 600 rpm with a temperature of 47 °C. The reacted solution was centrifuged at 10,000 rpm, the precipitate was collected and washed three times with ultrapure water with the aim to remove the residual sodium selenite. After that, the precipitate was dissolved in 10 mL of ultrapure water. Next, low molecular weight chitosan (0.1 g) was dissolved in 10 mL of 3% acetic acid solution, after complete dissolution, the low molecular weight chitosan aqueous solution was added to the mixture from the previous step and stirred at room temperature without light for 1 h. The precipitate was centrifuged according to the above method, and the low molecular weight chitosan coated elemental selenium LCS-SeNPs were obtained after the precipitate was freeze-dried.

### 4.3. Characterization and Measurements

The particle size of LCS-SeNPs was measured using a Zetasizer_Nano_ZS90 particle analyzer (Malvern Instruments Limited, Malvern, UK). The valence states of LCS-SeNPs was analyzed using an X-ray photoelectron spectroscopy (XPS, ESCALAB 250Xi, Thermo Fisher Scientific, Waltham, MA, USA). The LCS-SeNPs were detected using an Energy Dispersive X-Ray Spectroscopy (EDX, Cambridge, MA, USA), and LCS-SeNPs were observed using a transmission electron microscope (TEX, Thermo Fisher Scientific, USA) operated at 200 kV.

### 4.4. Experimental Animals

All animal experiments were conducted within the animal laboratory of Zhejiang Ocean University and were approved by the animal committee of Zhejiang Ocean University. ICR mice with an average body weight of 20 ± 2 g were purchased from Shanghai Slake Experimental Animal Co., Ltd. (Shanghai, China) Before the formal experiment, all mice adapted to the new conditions with a temperature of 21 ± 2 °C and light conditions (12 light–dark cycle). During this time, they were allowed to obtain food and water freely. Mice were randomly divided into the following three groups (*n* = 8 for each group): control group (normal treatment); the DSS group, (DSS-induced colitis); the LCS-SeNPs group (LCS-SeNPs (4 mg/kg/day)). In this experiment, 3.5% (*w*/*v*) of DSS was used to induce colitis. During the formal experiment, the mice in the control group drank pure water normally. Mice in the DSS group and the LCS-SeNPs group were provided water with 3.5% of DSS for 7–21 days. At the beginning of the experiment, mice in the LCS-SeNPs group were treated with LCS-SeNPs by gavage with a daily dose of 4 mg/kg/day until the end of the experiment; meanwhile, mice in the control and the DSS group were treated with pure water by gavage with a daily dose of 4 mg/kg/day.

The body weight of each mouse was recorded once a day and at the conclusion of day 21, mice were sacrificed. The serum was collected and centrifuged (4000 rpm, 4 °C for 15 min). The colon was removed immediately and its length was measured, after that, the colon was exposed to 4% paraformaldehyde for histological assessment. The remaining colon samples were rapidly frozen and stored at −80 °C for further analysis.

### 4.5. Histopathological Analysis

The collected colon was washed with PBS (pH 7.2–7.4) buffer three times, then were fixed with 4% paraformaldehyde solution for 24 h at room temperature (25 °C). Finally, the colon samples were sent for examination, embedded in paraffin, sectioned and stained with hematoxylin and eosin (H&E) and photographed and observed for any histopathological changes.

### 4.6. Detection of Cytokines in Serum

The blood of the eyeball was collected quickly before the mice were sacrificed and collected in a sterile dry EP tube. After standing overnight, the serum was centrifuged at 4 °C at 3000 rpm/min for 10 min. The serum was separated and stored in a refrigerator at −80 °C for subsequent detection. Serum levels of inflammatory factors (TNF-α and IL-6), intestinal injury levels (DAO and LPS) and antioxidant factors (SOD and GSH-Px) in mice were detected by a kit, and operations were carried out according to the kit instructions.

### 4.7. Detection of Cytokines Levels in Colon

In this experiment, 1 mL of normal saline was taken from 0.1 g of colon tissue, ground in a tissue homogenizer, centrifuged for 10 min at 4 °C and 3000 rpm/min, and the supernatant was separated and stored in −80 °C for later use. The levels of glutathione peroxidase (GSH-Px), malondialdehyde (MDA), interleukin-6 (IL-6) and lipopolysaccharide (LPS) in mouse tissue homogenate were detected using the relevant kits, and specific operations were carried out according to the kit instructions.

### 4.8. Determination of Tight Junction Protein mRNA Expression in Colon

Real-time quantitative PCR was performed to evaluate the relative expression levels of tight junction proteins ZO-1 and occludin. Total RNA was extracted from mouse colon tissue using cold TRIzol reagent (Ambion, Carlsbad, USA). The mass and concentration of the total RNA were quantified by spectrophotometry at 260 nm and 280 nm. The first cDNA strand was synthesized using the PrimeScript 1st Strand cDNA Synthesis Kit (Takara, Dalian, Japan). Primers were purchased from Shanghai Sangong Biotechnology Co., Ltd. (Shanghai, China) and listed in Table 1. Quantitative RT-PCR in Applied Biosystems ViiATM7 Real-Time PCR System (ViiATM7 Real-Time PCR System, Thermo Fisher Scientific, New York, NY, USA) using SYBR Green PCR Master Mix (Takara, Dalian, Japan). Real-time PCR conditions were 95 °C, 30 s, 95 °C, 5 s, 60 °C, 34 s for 40 cycles, and the melting-curve analysis was performed after each reaction to ensure a specific reaction. Relative expression was calculated by the 2^−∆∆Ct^ method.

### 4.9. Western Blotting Analysis

RIPA pyrolysis buffer is used to prepare tissue lysates. Protein concentration was decided utilizing the BCA protein assay kit. The protein samples were isolated by SDS-PAGE (10%) and transferred to a 0.45 μm polyvinylidene fluoride (PVDF) membrane (Merck Millipore, Burlington, MA, USA). After sealing 5% skim milk at room temperature for 1 h, the membrane was incubated with a primary antibody at 4 °C overnight. Then the membrane was washed with TBS 3 times for 5 min each time and incubated with HRP conjugated secondary antibody at room temperature for 1 h. Then the membrane was washed with TBST 3 times for 5 min each time, and ECL reagent (Beyotime, Shanghai, China) was added for chemiluminescence imaging. The band density was determined by Image J software, and the protein ratio detected was normalized for β-actin.

### 4.10. Statistical Analysis

Data were presented as mean ± standard error (SEM). The statistical significance was analyzed by one-way analysis of variance (ANOVA) and Tukey’s test with GraphPad Prism 8 software. A value of *p* < 0.05 was considered as significant.

## 5. Conclusions

In the present study, we develop a relatively uniform nano-selenium LCS-SeNPs with an average diameter of 198 nm. LCS-SeNPs alleviate DSS-induced colitis in mice, probably because of its combined favorable effects, which inhibits pro-inflammatory cytokines (IL-6 and TNF-α) and the production of oxidative index MDA, promotes the production of GSH-Px resulting in the restoration of intestinal permeability. Moreover, LCS-SeNPs depress oxidative stress by inhibiting the Nrf2 signaling pathway, reducing inflammation, and protect the intestinal barrier function by inhibiting the NF-κB signaling pathway.

## Figures and Tables

**Table 1:** Primers of target genes for RT-PCR.

Gene	Gene Accession Number	Primer Sequence 5’-3’	Product Size (bp)
ZO-1	XM_006540785	F: TACCTCTTGAGCCTTGAACTT R: CGTGCTGATGTGCCATAATA	248
Occludin	XM_011244634	F: GCCCAGGCTTCTGGATCTATGT R: GGGGATCAACCACACAGTAGTGA	124
β-actin	NM_007393	F: AGTGTGACGTTGACATCCGT R: GCAGCTCAGTAACAGTCCGC	298

## References

[1] Gheorghe C., Pascu O., Gheorghe L., Iacob R., Dumitru E., Tantau M., Vadan R., Goldis A., Balan G., Iacob S. (2004). Epidemiology of inflammatory bowel disease in adults who refer to gastroenterology care in Romania: A multicentre study. Eur. J. Gastroenterol Hepatol..

[2] Szigethy E., McLafferty L., Goyal A. (2010). Inflammatory bowel disease. Child Adolesc. Psychiatr. Clin. N. Am..

[3] Guan Q. (2019). A Comprehensive Review and Update on the Pathogenesis of Inflammatory Bowel Disease. J. Immunol. Res..

[4] Zou P., Yang X., Wang J., Li Y., Yu H., Zhang Y., Liu G. (2016). Advances in characterisation and biological activities of chitosan and chitosan oligosaccharides. Food Chem..

[5] Garcia-Fuentes M., Alonso M.J. (2012). Chitosan-based drug nanocarriers: Where do we stand?. J. Control. Release.

[6] Luo Y., Zhang B., Cheng W.H., Qin W. (2010). Preparation, characterization and evaluation of selenite-loaded chitosan/TPP nanoparticles with or without zein coating. Carbohydr. Polym..

[7] Mattaveewong T., Wongkrasant P., Chanchai S., Pichyangkura R., Chatsudthipong V., Muanprasat C. (2016). Chitosan oligosaccharide suppresses tumor progression in a mouse model of colitis-associated colorectal cancer through AMPK activation and suppression of NF-kappa B and mTOR signaling. Carbohydr. Polym..

[8] Benchamas G., Huang G.L., Huang S.Y., Huang H.L. (2021). Preparation and biological activities of chitosan oligosaccharides. Trends Food Sci. Technol..

[9] Zhang G., Jia P., Cheng G., Jiao S., Ren L., Ji S., Hu T., Liu H., Du Y. (2017). Enhanced immune response to inactivated porcine circovirus type 2 (PCV2) vaccine by conjugation of chitosan oligosaccharides. Carbohydr. Polym..

[10] Kieliszek M. (2019). Selenium-Fascinating Microelement, Properties and Sources in Food. Molecules.

[11] Navarro-Alarcon M., Cabrera-Vique C. (2008). Selenium in food and the human body: A review. Sci. Total Environ..

[12] Triggiani V., Tafaro E., Giagulli V.A., Sabbà C., Resta F., Licchelli B., Guastamacchia E. (2009). Role of iodine, selenium and other micronutrients in thyroid function and disorders. Endocr. Metab. Immune Disord. Drug Targets.

[13] Wu H.Y., Xia Y.M., Chen X.S. (1995). Selenium deficiency and thyroid hormone metabolism and function. Sheng Li Ke Xue Jin Zhan.

[14] Brown K.M., Arthur J.R. (2001). Selenium, selenoproteins and human health: A review. Public Health Nutr..

[15] Rayman M.P. (2000). The importance of selenium to human health. Lancet.

[16] Li T.Y., Xu H.P. (2020). Selenium-Containing Nanomaterials for Cancer Treatment. Cell Rep. Phys. Sci..

[17] Razaghi A., Poorebrahim M., Sarhan D., Björnstedt M. (2021). Selenium stimulates the antitumour immunity: Insights to future research. Eur. J. Cancer.

[18] Kong L., Yuan Q., Zhu H., Li Y., Guo Q., Wang Q., Bi X., Gao X. (2011). The suppression of prostate LNCaP cancer cells growth by Selenium nanoparticles through Akt/Mdm2/AR controlled apoptosis. Biomaterials.

[19] Wang L., Li X., Wang B. (2018). Synthesis, characterization and antioxidant activity of selenium modified polysaccharides from Hohenbuehelia serotina. Int. J. Biol. Macromol..

[20] Skalickova S., Milosavljevic V., Cihalova K., Horky P., Richtera L., Adam V. (2017). Selenium nanoparticles as a nutritional supplement. Nutrition.

[21] Fairweather-Tait S.J., Bao Y., Broadley M.R., Collings R., Ford D., Hesketh J.E., Hurst R. (2011). Selenium in human health and disease. Antioxid. Redox Signal..

[22] Geerling B.J., Badart-Smook A. (2000). Comprehensive nutritional status in recently diagnosed patients with inflammatory bowel disease compared with population controls. Eur. J. Clin. Nutr..

[23] Barrett C.W., Kshipra S., Motley A.K., Lintel M.K., Elena M., Bradley A.M., Ning W., Poindexter S.V., Bobak P., Reddy V.K. (2013). Dietary Selenium Deficiency Exacerbates DSS-Induced Epithelial Injury and AOM/DSS-Induced Tumorigenesis. PLoS ONE.

[24] Liljana G., Bishop K.S., Yeo H.D., Morgan A.R., Fraser A.G., Jiun L.W., Nishi K., Bobbi C., Ferguson L.R. (2012). Selenium, Selenoprotein Genes and Crohn’s Disease in a Case-Control Population from Auckland, New Zealand. Nutrients.

[25] Yang H., Zhu C., Yuan W., Wei X., Liu C., Huang J., Yuan M., Wu Y., Ling Q., Hoffmann P.R. (2022). Mannose-rich Oligosaccharides-functionalized selenium nanoparticles mediates Macrophage reprogramming and inflammation resolution in ulcerative colitis. Chem. Eng. J..

[26] Sakr T.M., Korany M., Katti K.V. (2018). Selenium nanomaterials in biomedicine—An overview of new opportunities in nanomedicine of selenium. J. Drug Deliv. Sci. Technol..

[27] Huang Y., Su E., Ren J., Qu X. (2021). The recent biological applications of selenium-based nanomaterials. Nano Today.

[28] Khurana A., Tekula S., Saifi M.A., Venkatesh P., Godugu C. (2019). Therapeutic applications of selenium nanoparticles. Biomed. Pharmacother..

[29] Ensign L.M., Cone R., Hanes J. (2012). Oral drug delivery with polymeric nanoparticles: The gastrointestinal mucus barriers. Adv. Drug Deliv. Rev..

[30] Agrawal U., Sharma R., Gupta M., Vyas S.P. (2014). Is nanotechnology a boon for oral drug delivery?. Drug Discov. Today.

[31] Chaudhary S., Umar A., Mehta S.K. (2016). Selenium nanomaterials: An overview of recent developments in synthesis, properties, and potential applications. Prog. Mater. Sci..

[32] Bajaj M., Winter J. (2014). Se (IV) triggers faster Te (IV) reduction by soil isolates of heterotrophic aerobic bacteria: Formation of extracellular SeTe nanospheres. Microb. Cell Factories.

[33] Bhattacharjee A., Basu A., Bhattacharya S. (2019). Selenium nanoparticles are less toxic than inorganic and organic selenium to mice in vivo. Nucleus.

[34] Huang Z., Rose A.H., Hoffmann P.R. (2012). The role of selenium in inflammation and immunity: From molecular mechanisms to therapeutic opportunities. Antioxid. Redox Signal..

[35] Versantvoort C.H., Withoff S., Broxterman H.J., Kuiper C.M., Scheper R.J., Mulder N.H., de Vries E.G. (1995). Resistance-associated factors in human small-cell lung-carcinoma GLC4 sub-lines with increasing adriamycin resistance. Int. J. Cancer..

[36] Liao G., Tang J., Wang D., Zuo H., Zhang Q., Liu Y., Xiong H. (2020). Selenium nanoparticles (SeNP) have potent antitumor activity against prostate cancer cells through the upregulation of miR-16. World J. Surg..

[37] Gao F., Zhao J., Liu P., Ji D., Zhang L., Zhang M., Li Y., Xiao Y. (2020). Preparation and in vitro evaluation of multi-target-directed selenium-chondroitin sulfate nanoparticles in protecting against the Alzheimer’s disease. Int. J. Biol. Macromol..

[38] Ren S.-X., Zhang B., Lin Y., Ma D.-S., Yan H. (2019). Selenium nanoparticles dispersed in phytochemical exert anti-inflammatory activity by modulating catalase, GPx1, and COX-2 gene expression in a rheumatoid arthritis rat model. Med. Sci. Monit..

[39] Zhai X., Zhang C., Zhao G., Stoll S., Ren F., Leng X. (2017). Antioxidant capacities of the selenium nanoparticles stabilized by chitosan. J. Nanobiotechnol..

[40] Zhang X., Yan H., Ma L., Zhang H., Ren D.F. (2020). Preparation and characterization of selenium nanoparticles decorated by Spirulina platensis polysaccharide. J. Food Biochem..

[41] Desai M.P., Labhasetwar V., Walter E., Levy R.J., Amidon G.L. (1997). The mechanism of uptake of biodegradable microparticles in Caco-2 cells is size dependent. Pharm. Res..

[42] Wang H., Xu M.Z., Liang X.Y., Nag A., Zeng Q.Z., Yuan Y. (2023). Fabrication of food grade zein-dispersed selenium dual-nanoparticles with controllable size, cell friendliness and oral bioavailability. Food Chem..

[43] Zeng S., Ke Y., Liu Y., Shen Y., Zhang L., Li C., Liu A., Shen L., Hu X., Wu H. (2018). Synthesis and antidiabetic properties of chitosan-stabilized selenium nanoparticles. Colloids Surf B Biointerfaces.

[44] Qiu W.Y., Wang Y.Y., Wang M., Yan J.K. (2018). Construction, stability, and enhanced antioxidant activity of pectin-decorated selenium nanoparticles. Colloids Surf B Biointerfaces.

[45] Tang L., Luo X., Wang M., Wang Z., Guo J., Kong F., Bi Y. (2021). Synthesis, characterization, in vitro antioxidant and hypoglycemic activities of selenium nanoparticles decorated with polysaccharides of *Gracilaria lemaneiformis*. Int. J. Biol. Macromol..

[46] Khiralla G., Elhariry H., Selim S.M. (2020). Chitosan-stabilized selenium nanoparticles attenuate acrylamide-induced brain injury in rats. J. Food Biochem..

[47] Shao C., Yu Z., Luo T., Zhou B., Song Q., Li Z., Yu X., Jiang S., Zhou Y., Dong W. (2022). Chitosan-Coated Selenium Nanoparticles Attenuate PRRSV Replication and ROS/JNK-Mediated Apoptosis in vitro. Int. J. Nanomed..

[48] Lara H.H., Guisbiers G., Mendoza J., Mimun L.C., Vincent B.A., Lopez-Ribot J.L., Nash K.L. (2018). Synergistic antifungal effect of chitosan-stabilized selenium nanoparticles synthesized by pulsed laser ablation in liquids against Candida albicans biofilms. Int. J. Nanomed..

[49] Bai K., Hong B., Huang W., He J. (2020). Selenium-Nanoparticles-Loaded Chitosan/Chitooligosaccharide Microparticles and Their Antioxidant Potential: A Chemical and In Vivo Investigation. Pharmaceutics..

[50] Song X., Chen Y., Sun H., Liu X., Leng X. (2021). Physicochemical stability and functional properties of selenium nanoparticles stabilized by chitosan, carrageenan, and gum Arabic. Carbohydr. Polym..

[51] Liu Y., Huang W., Han W., Li C., Zhang Z., Hu B., Chen S., Cui P., Luo S., Tang Z. (2021). Structure characterization of Oudemansiella radicata polysaccharide and preparation of selenium nanoparticles to enhance the antioxidant activities. LWT.

[52] Cai J., Liu J., Fan P., Dong X., Zhu K., Liu X., Zhang N., Cao Y. (2021). Dioscin prevents DSS-induced colitis in mice with enhancing intestinal barrier function and reducing colon inflammation. Int. Immunopharmacol..

[53] Talapphet N., Palanisamy S., Li C., Ma N., Prabhu N.M., You S. (2021). Polysaccharide extracted from *Taraxacum platycarpum* root exerts immunomodulatory activity via MAPK and NF-κB pathways in RAW264.7 cells. J. Ethnopharmacol..

[54] Yarley O.P.N., Kojo A.B., Zhou C., Yu X., Gideon A., Kwadwo H.H., Richard O. (2021). Reviews on mechanisms of in vitro antioxidant, antibacterial and anticancer activities of water-soluble plant polysaccharides. Int. J. Biol. Macromol..

[55] Wang H., Zhang J., Yu H. (2007). Elemental selenium at nano size possesses lower toxicity without compromising the fundamental effect on selenoenzymes: Comparison with selenomethionine in mice. Free. Radic. Biol. Med..

[56] Zhang J.S., Gao X.Y., Zhang L.D., Bao Y.P. (2001). Biological effects of a nano red elemental selenium. Biofactors.

[57] Liu Y., Zeng S., Liu Y., Wu W., Shen Y., Zhang L., Li C., Chen H., Liu A., Shen L. (2018). Synthesis and antidiabetic activity of selenium nanoparticles in the presence of polysaccharides from *Catathelasma ventricosum*. Int. J. Biol. Macromol..

[58] Kuo W.-T., Zuo L., Odenwald M.A., Madha S., Singh G., Gurniak C.B., Abraham C., Turner J.R. (2021). The Tight Junction Protein ZO-1 Is Dispensable for Barrier Function but Critical for Effective Mucosal Repair. Gastroenterology.

[59] Martini E., Krug S.M., Siegmund B., Neurath M.F., Becker C. (2017). Mend Your Fences: The Epithelial Barrier and its Relationship With Mucosal Immunity in Inflammatory Bowel Disease. Cell. Mol. Gastroenterol..

[60] Camilleri M., Madsen K., Spiller R., Greenwood-Van Meerveld B., Verne G.N. (2012). Intestinal barrier function in health and gastrointestinal disease. Neurogastroenterol. Motil..

[61] Turner J.R. (2009). Intestinal mucosal barrier function in health and disease. Nat. Rev. Immunol..

[62] Hayes J.D., McMahon M. (2009). NRF2 and KEAP1 mutations: Permanent activation of an adaptive response in cancer. Trends Biochem Sci..

[63] Zhu L.H., Zhao K.L., Chen X.L., Xu J.X. (2012). Impact of weaning and an antioxidant blend on intestinal barrier function and antioxidant status in pigs. J. Anim. Sci..

[64] Vachharajani T.J., Work J., Issekutz A.C., Granger D.N. (2000). Heme oxygenase modulates selectin expression in different regional vascular beds. Am. J. Physiol. Heart Circ. Physiol..

[65] Reszka E., Wieczorek E., Jablonska E., Janasik B., Fendler W., Wasowicz W. (2015). Association between plasma selenium level and Nrf2 target genes expression in humans. J. Trace Elem. Med. Biol..

[66] Alves Júnior E.B., de Oliveira Formiga R., de Lima Serafim C.A., Cristina Araruna M.E., de Souza Pessoa M.L., Vasconcelos R.C., de Carvalho T.G., de Jesus T.G., Araújo A.A., de Araujo Junior R.F. (2020). Estragole prevents gastric ulcers via cytoprotective, antioxidant and immunoregulatory mechanisms in animal models. Biomed. Pharmacother..

[67] Li H., Shen L., Lv T., Wang R., Zhang N., Peng H., Diao W. (2019). Salidroside attenuates dextran sulfate sodium-induced colitis in mice via SIRT1/FoxOs signaling pathway. Eur. J. Pharmacol..

[68] Lei L., Yang J., Zhang J., Zhang G. (2021). The lipid peroxidation product EKODE exacerbates colonic inflammation and colon tumorigenesis. Redox Biol..

[69] Shi X.D., Tian Y.Q., Wu J.L., Wang S.Y. (2021). Synthesis, characterization, and biological activity of selenium nanoparticles conjugated with polysaccharides. Crit. Rev. Food. Sci. Nutr..

[70] Checker R., Patwardhan R.S., Sharma D., Menon J., Thoh M. (2012). Schisandrin B exhibits anti-inflammatory activity through modulation of the redox-sensitive transcription factors Nrf2 and NF-κB. Free. Radic. Biol. Med..

[71] Mitchell J.P., Carmody R.J. (2018). NF-κB and the Transcriptional Control of Inflammation. Int. Rev. Cell. Mol. Biol..

